# A novel anoikis-related gene signature predicts prognosis in patients with head and neck squamous cell carcinoma and reveals immune infiltration

**DOI:** 10.3389/fgene.2022.984273

**Published:** 2022-08-26

**Authors:** Hao Chi, Puyu Jiang, Ke Xu, Yue Zhao, Bingyu Song, Gaoge Peng, Bingsheng He, Xin Liu, Zhijia Xia, Gang Tian

**Affiliations:** ^1^ Clinical Medical College, Southwest Medical University, Luzhou, China; ^2^ Department of Otorhinolaryngology-Head and Neck Surgery, Shanghai Ninth People’s Hospital, Shanghai Jiao Tong University School of Medicine, Shanghai, China; ^3^ Department of Oncology, Chongqing General Hospital, Chongqing, China; ^4^ Department of Breast Surgery, The Cancer Hospital of the University of Chinese Academy of Sciences (Zhejiang Cancer Hospital), Hangzhou, China; ^5^ Department of General, Visceral, and Transplant Surgery, Ludwig-Maximilians-University Munich, Munich, Germany; ^6^ Department of Plastic Surgery, Xijing Hospital, Fourth Military Medical University, Xi’an, China; ^7^ Department of Laboratory Medicine, The Affiliated Hospital of Southwest Medical University, Luzhou, China

**Keywords:** anoikis, model, bioinformatics, prognosis, HNSCC

## Abstract

**Background:** Head and neck squamous cell carcinoma (HNSCC) is a highly aggressive disease with a poor prognosis for advanced tumors. Anoikis play a key role in cancer metastasis, facilitating the detachment and survival of cancer cells from the primary tumor site. However, few studies have focused on the role of anoikis in HNSC, especially on the prognosis.

**Methods:** Anoikis-related genes (ANRGs) integrated from Genecards and Harmonizome portals were used to identify HNSCC subtypes and to construct a prognostic model for HNSCC patients. Also, we explored the immune microenvironment and enrichment pathways between different subtypes. Finally, we provide clinical experts with a novel nomogram based on ANRGs, with DCA curves indicating the potential clinical benefit of the model for clinical strategies.

**Results:** We identified 69 survival-related HNSCC anoikis-related DEGs, from which 7 genes were selected to construct prognostic models. The prognostic risk score was identified as an independent prognostic factor. Functional analysis showed that these high and low risk groups had different immune status and drug sensitivity. Next risk scores were combined with HNSCC clinicopathological features together to construct a nomogram, and DCA analysis showed that the model could benefit patients from clinical treatment strategies.

**Conclusion:** The predictive seven-gene signature and nomogram established in this study can assist clinicians in selecting personalized treatment for patients with HNSCC.

## Introduction

Currently, HNSCC is the sixth most common cancer worldwide and the most common malignancy occurring in the head and neck region (Sung et al., 2021), usually originating from the mucosal epithelium of the oral cavity, pharynx and larynx. Frequent loss or gain of chromosomal regions in HNSCC patients, making them characterized by genetic instability (Raj et al., 2022). Therefore, HNSCC progresses rapidly, and once distant metastases are detected a median survival of 3.3–3.9 months is predicted (Duprez et al., 2017). Unfortunately, however, distant metastases occur in up to 15% of patients. Therefore, there is an urgent need for more novel biomarkers to predict the prognosis of patients with early stage HNSCC so that clinical interventions can be taken in time to delay the progression of the disease.

Anoikis is a form of programmed cell death, which is essential for the survival of tumor cells after detachment from the extracellular matrix (ECM) (Xiao et al., 2019; Zhou et al., 2022). The generation of anoikis resistance in aggressive tumor cells has been identified as a key factor in tumor progression (Berezovskaya et al., 2005; Kim et al., 2012; Yu et al., 2022). However, few studies have focused on the association between the anoikis process and distant metastasis of HNSCC.

Therefore, in this study, we focused on exploring the prognostic value of ANRGs in HNSCC and developed a prognostic scoring model based on ANRGs. Further to explore the differences in tumor microenvironment of patients under this risk score typing.

## Materials and methods

### Gene expression and clinical data acquisition

Gene expression profiles of 270 HNSCC tissues and 504 HNSCC tissues with 44 normal adjacent tissues were obtained from Gene Expression Omnibus (GSE65858) data portal and The Cancer Genome Atlas (TCGA-HNSC) databases, respectively.

### Acquisition of anoikis-related genes

A total of 316 anoikis-related genes (ANRGs) were downloaded from the GeneCard database (Rebhan et al., 1997) (https://www.genecards.org/) and Harmonizome portals (Rouillard et al., 2016). Further, 253 differentially expressed genes (DEG) were identified in TCGA-HNSC cohort via the “limma” R package, comparing the expression of 316 ANRGs between tumor tissues and adjacent normal tissues.

### Consensus clustering

Consensus clustering was applied to identify distinct anoikis-related patterns relating to the expression of anoikis regulators by the k-means method. Thereafter, Uniform Manifold Approximation and Projection (UMAP) was used to validate the reliability of clustering with the R package “ggplot2”.

### Functional enrichment analysis

We downloaded “c2. cp.kegg.v7.4. symbols.gmt” from the MSigDB database to carry out GSVA analysis. The “GSVA” R package was used to perform GSVA enrichment analysis (Hanzelmann et al., 2013).

### Development and validation of prognostic signatures based on anoikis-related genes

Univariate Cox regression analysis was performed to screen for genes associated with survival, followed by least absolute shrinkage and selection operator (LASSO) regression analysis using the R package “glmnet”, and the penalty regularization parameter *λ* was determined by tenfold cross-validation. Subsequently, a multivariate Cox regression model was used to identify the central genes and calculate their corresponding coefficients. Seven ANRGs were selected to construct risk signatures based on the best lambda values and corresponding coefficients. The risk score of the new ANRG signature for each patient was calculated as follows. RiskScore = e^(…. Corresponding coefficient + …. + LTF expression), with Coefi and Expi representing the risk coefficient and expression level of each gene, respectively. Kaplan-Meier (KM) survival curves and time-dependent receiver operational feature (ROC) curve analyses were made to assess the predictive capacity of the model.

In summary, seven anoikis-related DEGs closely related to OS were identified using univariate Cox regression and LASSO analysis in GSE65858 cohort, and validated in TCGA-HNSC cohort.

### Relationship between risk score and immune cell infiltration

CIBERSORT and ssGSEA R scripts were used to quantify the relative proportion of infiltrating immune cells (Newman et al., 2015). We used CIBERSORT to estimate the proportion of immune cell types between the low-risk and high-risk groups. The sum of all estimated immune cell type scores in each sample equals 1. Meanwhile, spearman rank correlation analysis was applied to explore relationships between risk score values and immune infiltrating cells.

### Construction and evaluation of a predictive nomogram

Clinicopathological characteristics and risk scores were used to construct the nomogram. The calibration plot was performed for an internal validation to verify the accuracy. Time-C index was used to validate the predictive performance of the nomogram. Decision curve analysis (DCA) was performed to assess the clinical net benefit (Vickers et al., 2008).

### Tumor immune single cell hub database

The Tumor Immune Single-Cell Hub (TISCH; http://tisch.comp-genomics.org) is alarge-scale online database of single-cell RNA-seq focused on the TME (Sun et al., 2021). This database was used to systematically investigate the TME heterogeneity invarious data sets and cell types.

### Statistical analysis

Statistical analyses were performed using R software v4.1.3. *p*-values < 0.05 were considered statistically significant and FDR (false discovery rate) q < 0.05 was considered statistically significant.

## Results

### Identification of prognosis-related anoikis-related genes

**FIGURE 1:**
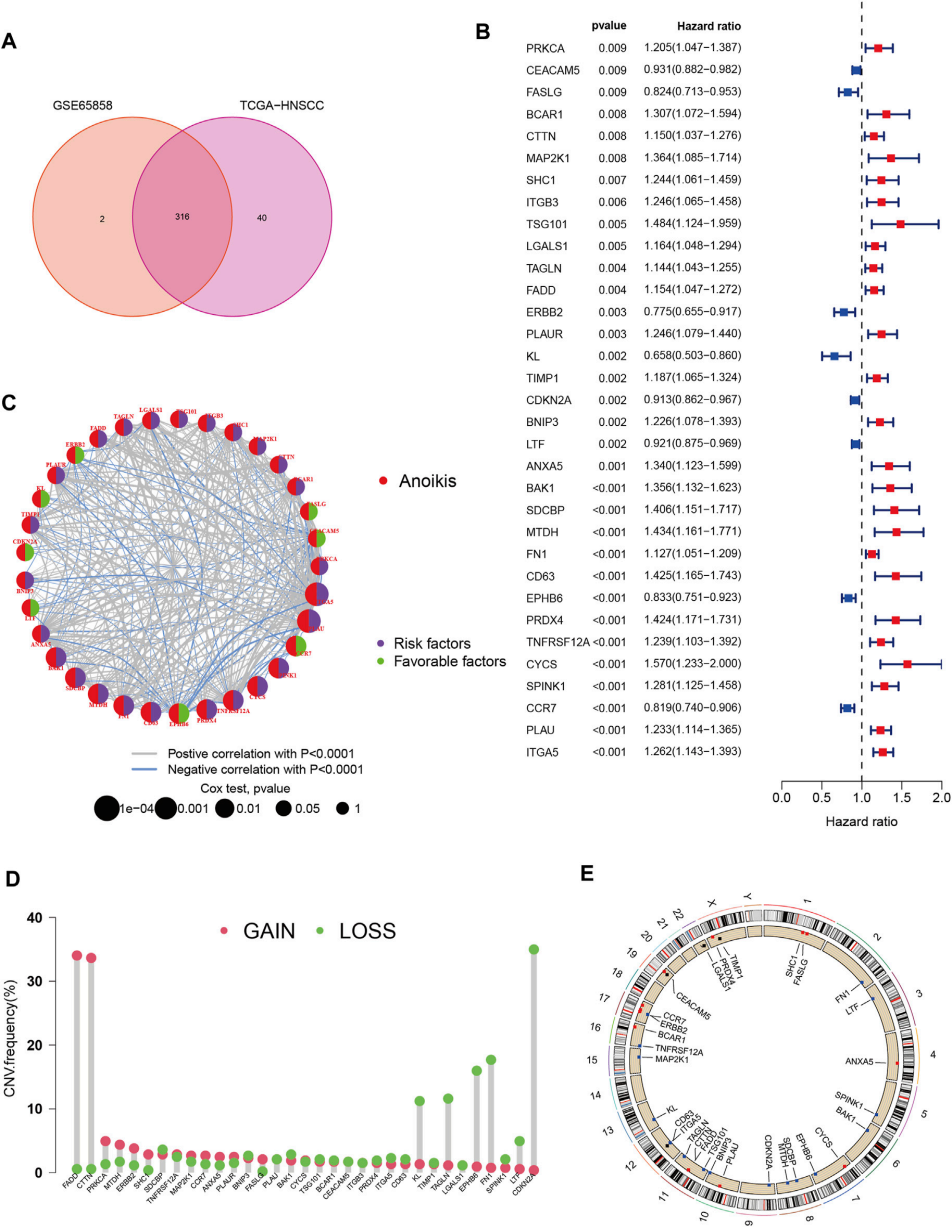
Characteristics and differences of anoikis-related regulators in HNSCC. **(A)** 316 anoikis-related genes identified from GSE65858 and TCGA-HNSCC cohort. **(B)** The forest plot shows the top 33 ANRGs (*p* < 0.01) *via* the univariate Cox regression analysis. **(C)** Network diagram showed the correlations between the top 33 ANRGs. **(D)** Copy number variations (CNVs) and of 33 ANRGs in TCGA-HNSCC. **(E)** Chromosome region and alteration of ANRGs.

A total of 358 anoikis-related genes were obtained from Genecards and Harmonizome portals. Then we got 316 ANRGs *via* Venn plot (Figure 1A). Then, 253 DEGs were found in HNSCC samples compared to normal adjacent tissues. We combined the TCGA-HNSC cohort with the GSE65858 cohort to remove the batch effect and obtained the new “HNSC-GSE65858” cohort, a total of 14,490 genes were retained. Univariate Cox regression analysis revealed 179 of 253 ANRGs were associated with survival (km < 0.05), of which 69 genes were statistically different (*p* < 0.05, km < 0.05). Univariate Cox regression analysis revealed 179 of 253 ANRGs were associated with survival (km < 0.05), of which 69 genes were statistically different (*p* < 0.05, km < 0.05). The forest plot shows the top 33 ANRGs (*p* < 0.01) (Figure 1B **)**. 24 genes were associated with poor prognosis except for FASLG, CEACAM5, ERBB2, KL, CDKN2A, LTF, EPHB6 and CCR7. Meanwhile, the network plot shows more clearly the relationship between the expression levels of the top 33 genes of rank (Figure 1C). As chromosome regions are frequently lost or gained in HNSCC patients (Raj et al., 2022), we downloaded CNV data from TCGA database to further explore how these anoikis-related genes are altered on chromosomes and where each is located on the chromosome (Figures 1D,E). As shown in Figure 1E, the most significant altered “gain” of FADD and CTTN was located on chromosome 11, while CDKN2A was mainly “loss” and located on chromosome 9.

### Using the 33 anoikis-related genes for the consistent clustering of Head and neck squamous cell carcinoma molecular subgroups

**FIGURE 2:**
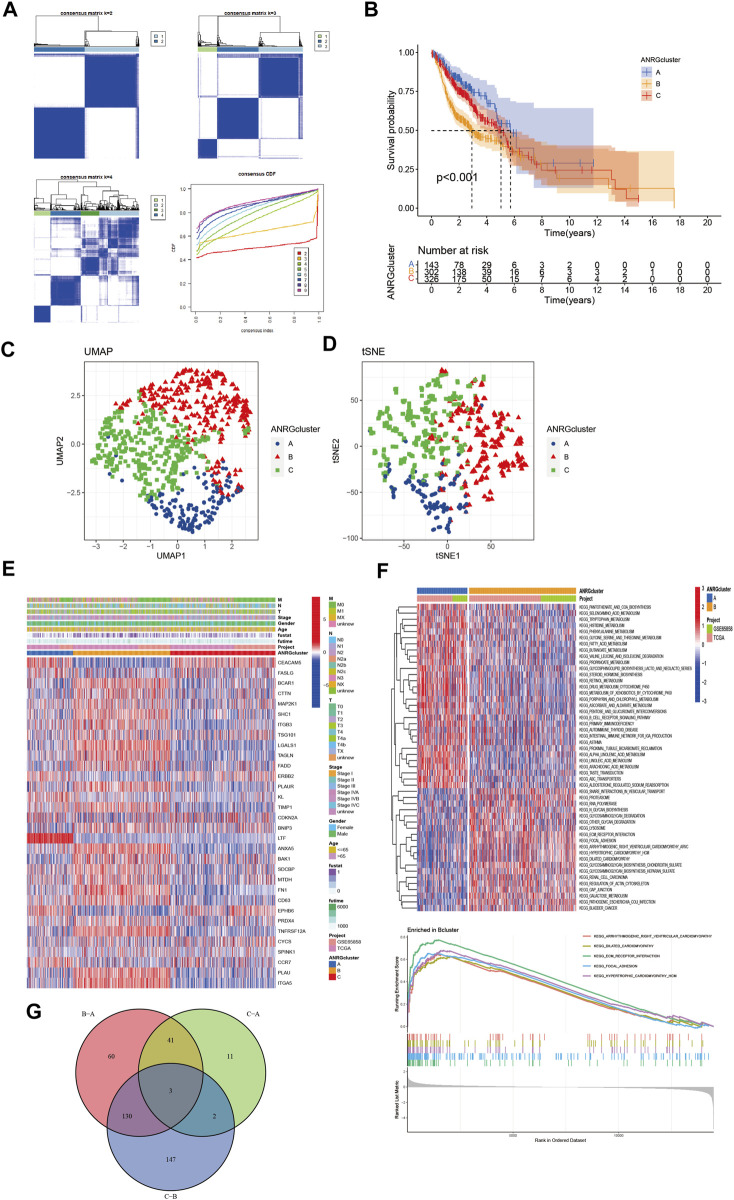
Subgroups of HNSCC related by anoikis-related genes. **(A)** Consensus matrix for *k* = 3 was obtained by applying consensus clustering. **(B)** Overall survival of three subtypes (*p* < 0.001) **(C,D)** UMAP and tSNE distinguished three subtypes based on the expression of ANRGs. **(E)** Heat map of ANRGs expression and corresponding clinicopathological features of three subtypes. **(F)** GSVA analysis focused on the differential enrichment of KEGG pathways between clusters B and A **(G)** Venn plot exhibited intersections between differential ANRGs.

To better understand the role of anoikis-related genes in HNCSS, we used 33 prognosis-related DEGs (*p* < 0.01) for Consensus Clustering by using the Consensus Cluster Plus R software package. As shown in Figure 2A, when *k* = 3, The cohort could be well classified into three subtypes. Overall survival analysis showed a significant difference in prognosis between the three subtypes (*p* < 0.01) (Figure 2B). UMAP and tSNE were used to test the accuracy of this clustering. The results showed that the three clustering subtypes could be well identified at *k* = 3 (Figures 2C,D). Heat map of ANRGs expression and corresponding clinicopathological features of three subtypes indicated LTF might be a factor of good prognosis. (Figure 2E). In addition to exploring the overall distribution of 33 ANRGs in the clusters, given the more significant differences between cluster B and cluster A, we applied the GSVA package to focus on the differential enrichment of KEGG pathways between clusters B and A (Figure 2F; Supplementary Figure S1). Cluster B, the least prognostic group, is mainly involved in the ECM receptor interaction and Focal adhesion pathways, which are key pathways for tumor cells to escape their original growth environment and colonize new anchor sites. Finally, the venn plot demonstrates the differential distribution of ANRGs among the three subtypes, with the three genes FAM3D, UPK18, and KRT19 differing significantly among the subgroups (Figure 2G).

### Gene expression and immune infiltration in three subtype clusters

**FIGURE 3:**
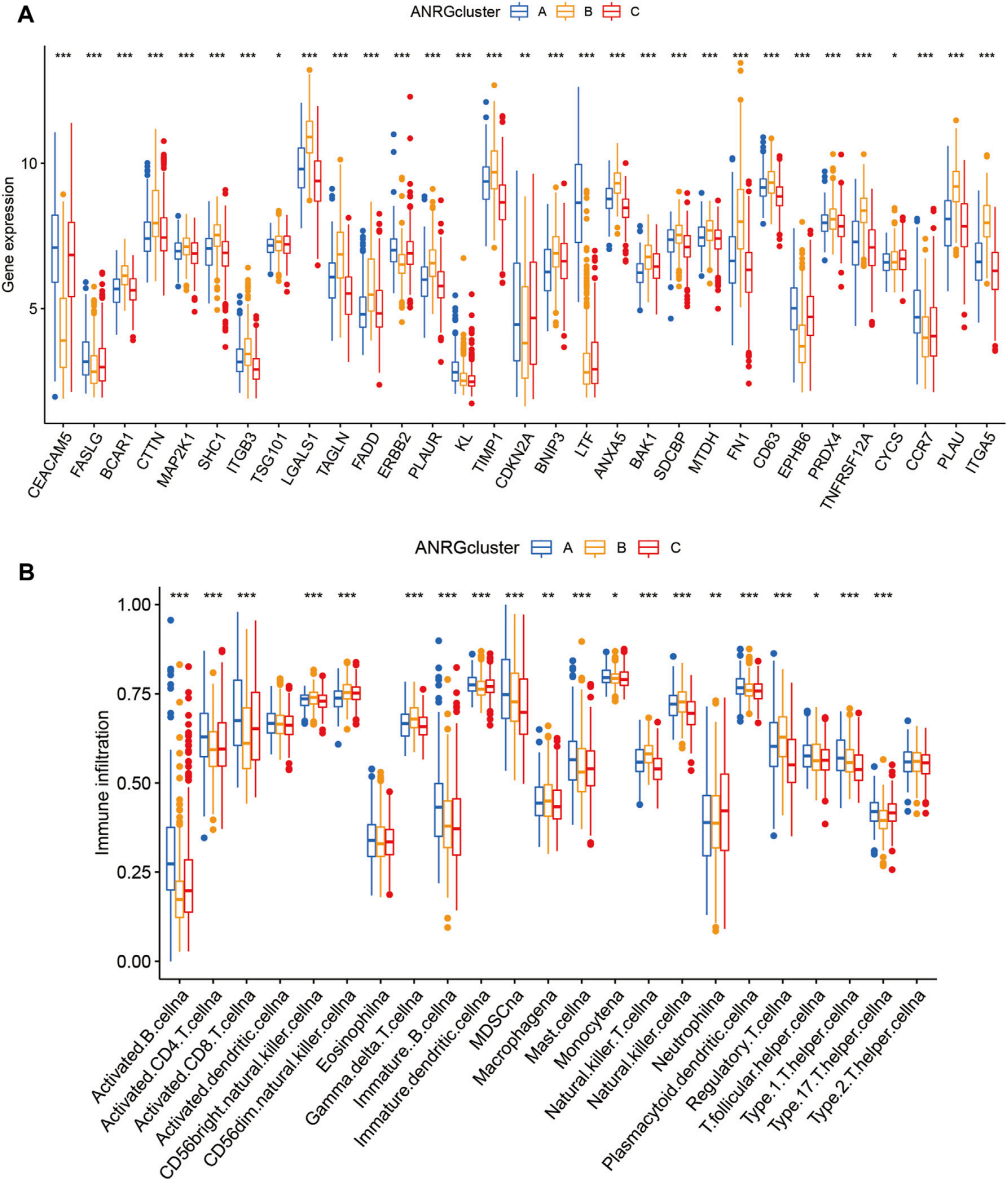
Gene expression and immune infiltration patterns in three subtype clusters. **(A)** ANRGs expression in three subtype clusters. **(B)** Immune infiltration patterns in three subtype clusters.

Boxplot was used to show the expression patterns of anoikis-related genes in the three subgroups. It can be seen that FASLG, CEACAM5, ERBB2, CDKN2A, LTF, EPHB6, and CCR7 are significantly less expressed in cluster B than in cluster A or cluster C; other significant ANRGs show high expression patterns. Because of the association with overall survival, these differential genes may be key molecules affecting the prognosis of HNSCC patients as well as potential targets for targeted therapy (Figure 3A). Besides, the level of immune cell infiltration also differed significantly (Figure 3B), with the proportion of activated CD4, CD8, and B lymphocytes in cluster B being significantly lower than in the other two subtypes.

### Construction and validation of a anoikis-related prognosis signature with good performance

**FIGURE 4:**
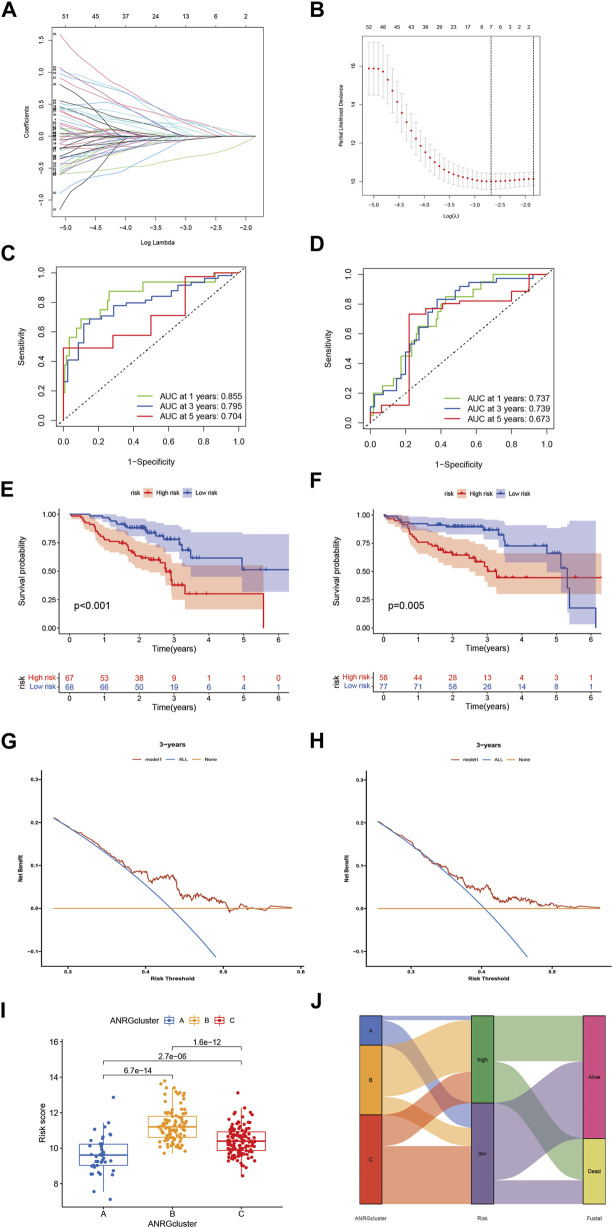
Identify anoikis-related prognosis signature. **(A)** LASSO analysis with 10-fold cross validation identified seven prognostic anoikis-related genes. **(B)** Coefficient profile plots of seven prognostic anoikis-related genes. **(C,D)** The time-dependent ROC curves for OS at 1-, 3-, and 5-years. **(E,F)** The K-M curves showed the different prognosis in subtype risk group. **(G)** DCA curve demonstrated the clinical net-benefit with constructed model in validation TCGA-HNSC cohort. **(I)** Risk score in 3 clusters established before **(J)** Alluvial diagram of subtype and living status.

To explore the clinical value of anoikis-related genes, we used 69 ANRGs (*p* < 0.05) involved in Lasso-penalized Cox analysis (Figures 4A,B). The final risk score based on the seven-ANRG signature is called “ANRGscore”, and the correlation coefficients are shown in Supplementary Supplementary Table S1. The prognostic index (PI) = (0.547 * expression level of MTDH) + (0.496* expression level of BAK1) + (0.422* expression level of PRDX4) + (0.368* expression level of IKZF3) + (0.326* expression level of FN1) - (0.45* expression level of ERBB2)—(0.157* expression level of LTF). The time-dependent ROC curves for OS at 1, 3, and 5 years exhibited good predictive performance with this model (Figures 4C,D). The K-M curves showed that patients in the high-risk group indicated a poorer prognosis, which was also observed in TCGA-HNSC validation cohort (Figures 4E,F). DCA curve in TCGA-cohort demonstrated that this model is a guide for clinical application and may benefit HNCSS patients both in OS and PFS (Figures 4G,H). The risk score was significantly different among the three previous subtypes (Figures 4I,J), Alluvial diagram showing the changes of anoikis-related clusters, ANRGscore and living status.

### Gene set enrichment analysis and immune activity with different risk score

**FIGURE 5:**
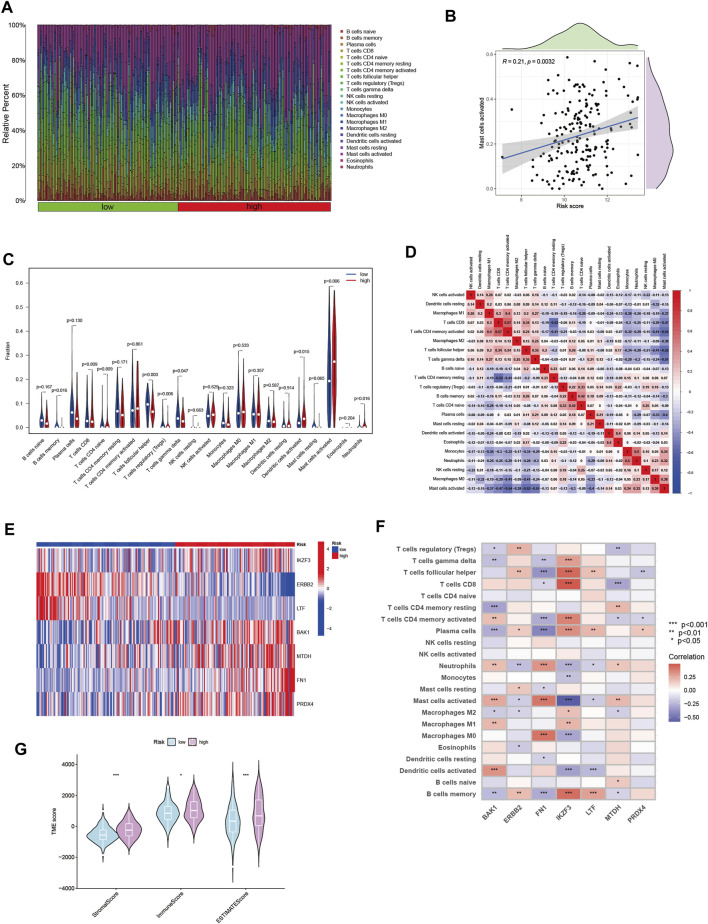
The immune microenvironment of HNSCC tissues at different risk score. **(A)** The relative proportion of infiltrating immune cells with different risk score. **(B)** The correlation analysis between risk score and the proportion of activated Mast cells in HNSCC tissues. **(C)** Immune cell component between high-risk group and low-risk group. **(D)** Correlation between immune cells. **(E)** Heatmap showing the expression patterns of seven hub ANRGs. **(F)** Correlation between immune cells and seven hub ANRGs. **(G)** Estimate score of the expression profile in high-risk group and low risk group.

The immune microenvironment plays an important role in the development of tumorigenesis and in the response to immunotherapy. To this end, we further explored the tumor microenvironment (TME) landscape of HNSCC patients in high- and low-risk groups. CIBERSORT R script was used to quantify the relative proportion of infiltrating immune cells. Firstly, ranking of HNSCC through risk score from lowest to highest, showing the proportion of different immune cells corresponding to risk score (Figure 5A). The proportion of activated Mast cells increased gradually with increasing risk score (*R* = 0.21) (Figure 5B). Moreover, activated Mast cells accounted for a larger proportion of the immune cell component in HNSCC patients (Figure 5C). This suggested that Mast cell activation may be an important reason for the poor prognosis of HNSCC patients (Gorzalczany and Sagi-Eisenberg, 2019). The correlation between immune cells in HNSCC patients may provide clues for a better understanding of the composition of the immune microenvironment in specific types of tumors (Figure 5D). The seven gene signature used to construct the ANRGscore model has different expression patterns between high and low risk groups and is closely associated with multiple immune cell infiltrations (Figures 5E,F). In addition, by estimatescore of the expression profile, we obtained the stromal score, immune score of the high and low risk groups (Figure 5G). In the end, with “pRRophetic” R package, we explored the potential sensitivity of high-risk group and low-risk group to clinical drugs (Supplementary Table S2; Supplementary Figure S2).

### Establishment of a prognostic nomogram for Head and neck squamous cell carcinoma patients

**FIGURE 6:**
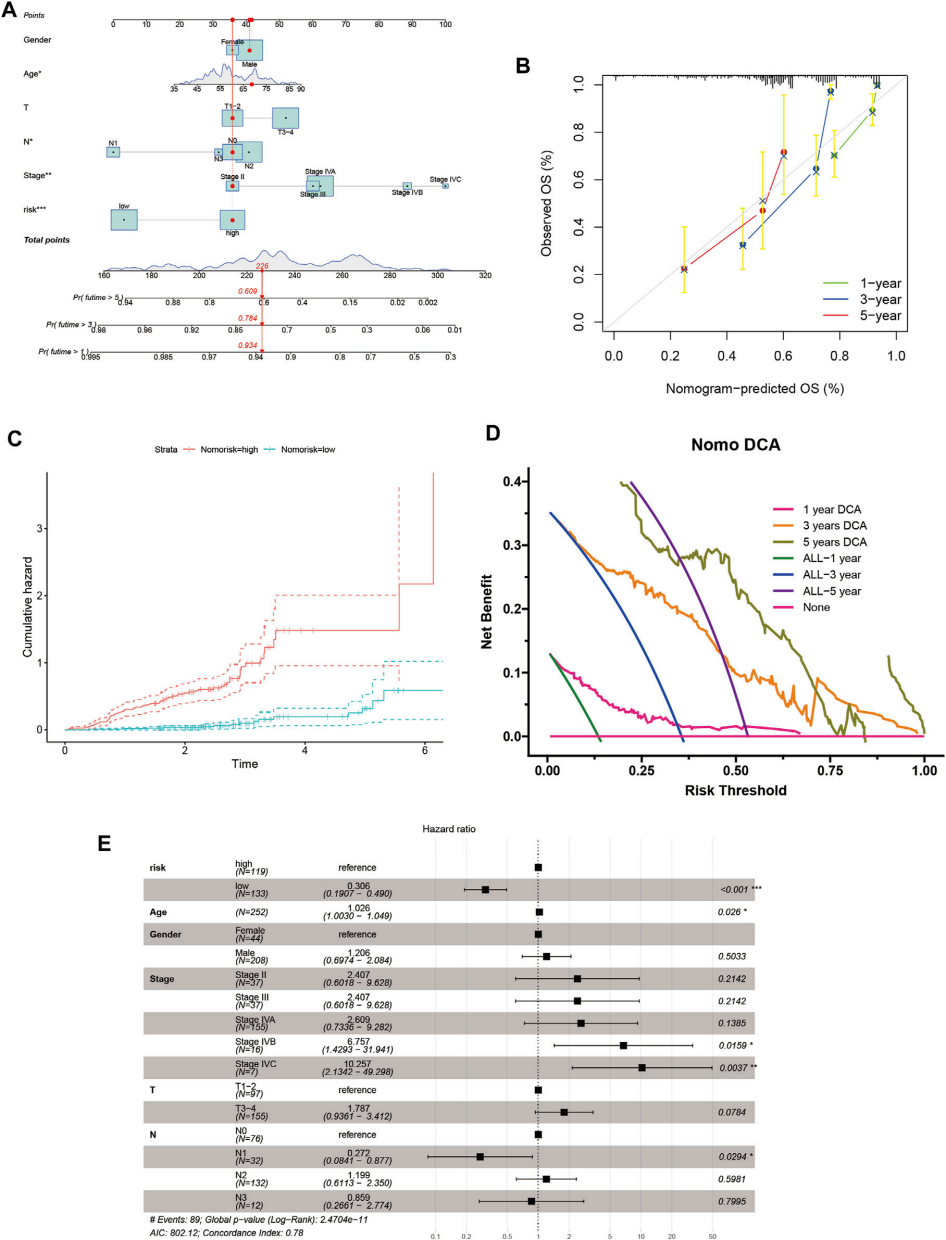
Nomogram for HNSCC patients. **(A)** Nomogram plot based on ANRGscore and clinicopathological factors. **(B)** Calibration plot for the validation of the nomogram. **(C)** Cumulative hazard curve represented the probability of survival over time progression. **(D)** DCA curves of the nomogram for 1-, three- and five- year OS in HNSCC patients **(E)** Forest plot summary of multivariable Cox regression analyses of the clinical features as well as risk score in HNSCC patients.

Considering the influence of clinicopathological factors on the prediction model, we combined the ANRGscore model with clinical information to construct the nomogram (Figure 6A). The calibration plot showed the validation of the nomogram (Figure 6B). The cumulative hazard curve also showed a progressive increase in overall survival risk for patients with high scores of HNSCC patients in the nomogram (Figure 6C). Decision Curve Analysis (DCA) is a simple method for evaluating clinical predictive models, diagnostic tests, and molecular markers, and is often used to clinically evaluate whether a strategy will benefit patients. The nomogram exhibited as a good method for predicting short- and long-term survival of HNSCC patients (Figure 6D). The forest plot shows that in the nomogram, riskScore and T stage are the main influencing factors (Figure 6E). These results suggest that the nomogram with risk scores based on ANRGs can be used as an effective method to predict patient prognosis in clinical practice.

### Correlation analysis of anoikis-related genes and Tumor immune microenvironment

**FIGURE 7:**
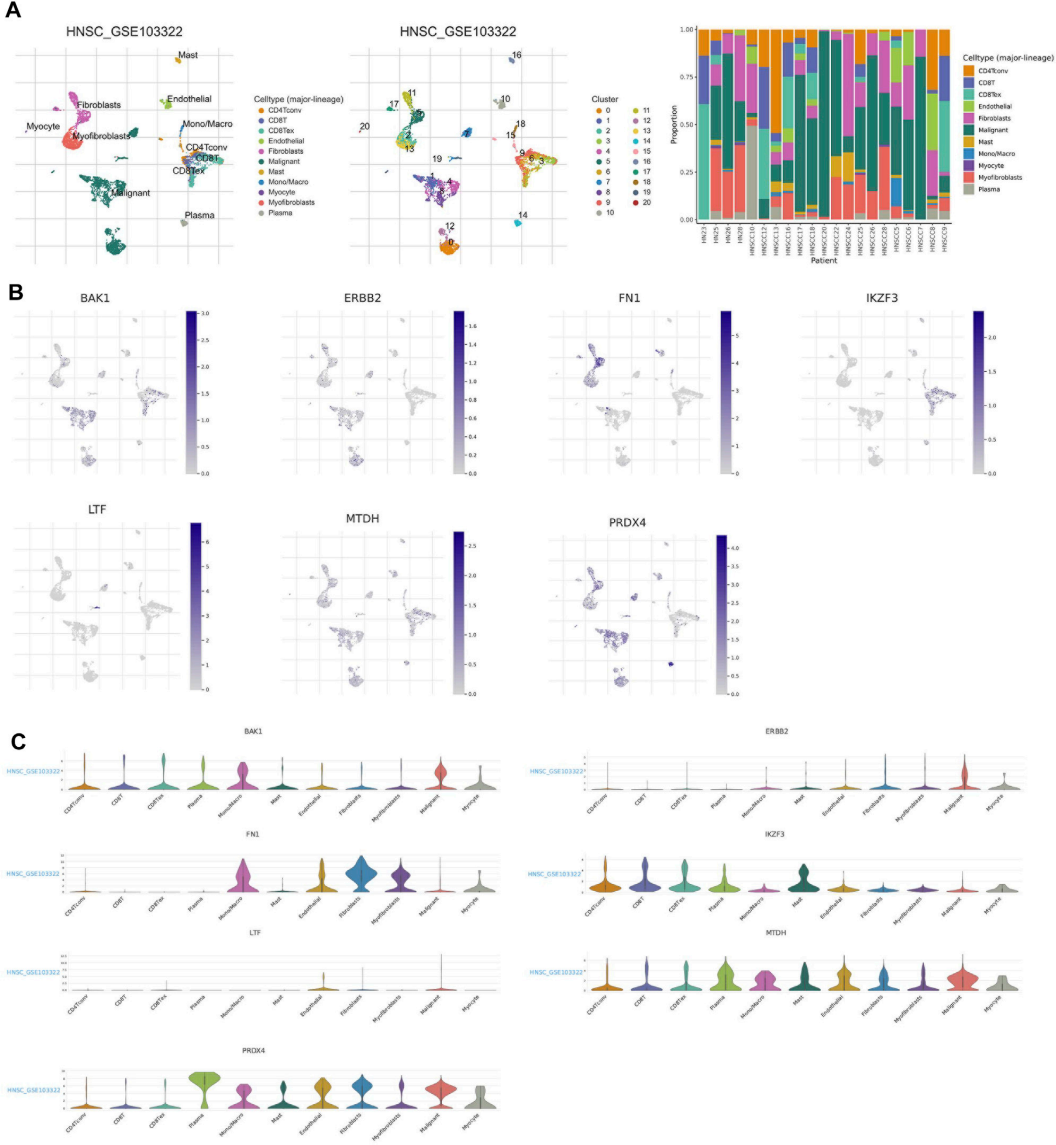
ANRGs Expression in HNSC TME-associated cells. **(A)** Annotation of all celltypes in GSE103322 and the percentage of each cell type **(B,C)** Percentages andexpressions of BAK1, ERBB2, FN1, IKZF3, LTF, MTDH, PRDX4.

We used the single cell dataset HNSC_GSE103322 from the TISCH database to analyze the expression of seven ANRGs in TME. In the GSE103322 dataset, there are 20 cell clusters and 11 medium cell types, and the distribution and number of various cell types are shown (Figure 7A). BAK1 is mainly expressed in malignant cells and immune cells (CD4Tconv, CD8T, and CD8Rex), while it is expressed at lower levels in Fibroblasts and Myofibroblasts. ERBB2 and FNA1 were expressed in malignant cells and stromal cells (Fibroblasts, Myofibroblasts and Myocyte), but almost not in immune cells. IKZF3 was only detected at expression levels in immune cells. Ltf was almost not detected in tme. MTDH and PRDX4 were expressed in tme High expression was found in a variety of cells, MTDH was mainly expressed in immune cells and PRDX4 was mainly expressed in malignant and stromal cells (Figures 7B,C).

## Discussion

Head and neck squamous cell carcinoma (HNSCC) is a highly aggressive disease (Bhat et al., 2021). The rapidly progressive disease makes it difficult to improve the prognosis of patients with aggressive HNSCC through a single targeted route or drug therapy in a timely manner (Johnson et al., 2020). Therefore, the construction of predictive models using metastasis-associated genes may provide important tools for early intervention. However, the number of such markers is not enough. Thus, there is an urgent need to screen for more biomarkers with high predictive performance to be included in the candidate list.

Anoikis is a programmed cell death that occurs when cells separate from the correct extracellular matrix, thereby disrupting the attachment of integrins (Taddei et al., 2012). It is a key mechanism for preventing dysplastic cells from growing or attaching to an inappropriate matrix (Li et al., 2021). Anoikis prevents detached epithelial cells from settling elsewhere and is therefore essential for tissue homeostasis and development (Bakir et al., 2020).

Normally, when normal epithelial cells detach, they lose critical survival factors and undergo a programmed cell death called anoikis (Nirmala and Lopus, 2020), while HNSCC metastatic tumor cells gain anoikis resistance, which allows them to begin curating away from the primary lesion (Braunholz et al., 2016; Liao et al., 2017; Shen et al., 2017). Owing of the fact that anchorage-dependent growth and epithelial-mesenchymal transition, two features associated with anoikis resistance, are critical steps in tumor progression and metastatic spread of cancer cells, anoikis dysregulation is now of particular interest to the scientific community (Talukdar et al., 2018; Corbet et al., 2020; Ye et al., 2020; Yoon and DeNicola, 2021; Yu et al., 2022). Multiple pathways can lead to the acquisition of anoikis resistance in HNSCC (Dey et al., 2015), and these highlight the concept of targeting anoikis-related genes to overcome HNSCC progression and metastasis (Kumar et al., 2010). Polygenic analysis reflects the complex interplay of various parameters affecting anoikis resistance in tumor pathology. Thus, this polygenic approach may allow characterization of tumor biology to support clinical decision making in the era of precision medicine in cancer.

In this study, we identified robust risk score features containing seven genes, namely IKZF3, BAK1, MTDH, FN1, PRDX4, ERBB2, and LTF. Previous studies have described certain associations between genes and the tumorigenesis and pathogenesis of cancer. For example, Xichuan Li et al. indicated that IKZF3 (Aiolos) could reduce the expression of many adhesion-related genes, thereby disrupting ECM integrity (Li et al., 2014). Similarly, *via* suppressing the expression level of PRDM1 in lung cancer, Aiolos promoted anoikis resistance and distant metastasis *in vivo* (Zhu et al., 2017). Besides, MiR-125b promotes PCa xenograft tumor growth by targeting pro-apoptotic genes such as BAK1 (Shi et al., 2011)。Lance S Terada et al. identified Aiolos as an epigenetic driver of lymphocyte mimicry in aggressive cancers, linking the development of immune cells to metastatic behavior (Terada and Liu, 2014). MTDH is overexpressed in hepatocellular carcinoma and is strongly associated with tumor metastasis. MTDH could induce autophagy, which leads to anoikis resistance and a key factor for metastasis (Zhu et al., 2020). Besides, MTDH-dependent anoikis resistance is activated by the PI3K/Akt pathway, and anoikis resistance is obtained by inhibiting caspase-3 activation and activating CXCR4 expression levels (Zhou et al., 2014), targeting MTDH can limit PDAC metastasis (Suzuki et al., 2017). Recently, a study suggested that FN1 promotes cellular aggregate formation conferring anoikis resistance to tumor cells (Han et al., 2021), and that pretreatment of exosomes with anti-FN1 antibodies attenuates the invasive ability of fibroblasts (Shafiq et al., 2021). PRDX4 is overexpressed in a variety of tumor tissues (Jia et al., 2019), and inhibits anoikis resistance through the β-catenin/ID2 pathway, thereby promoting the growth and metastasis of hepatocellular carcinoma cells (Wang et al., 2019). Lactoferrin (LTF) can induce anoikis in infected enterocytes (Sherman and Petrak, 2005), which may be a potential target to inhibit the development of metastasis in HNSCC. ERBB2 blocks anoikis in breast cancer cells by downregulating the pro-apoptotic proteins Perp and Bim in a Mek-dependent manner (Reginato et al., 2003; Khan et al., 2016)

Sample classification based on predefined gene expression profiles is a proven method. Borrowing from this approach, HNSCC patients were classified according to the expression of anoikis-related genes, which were expressed significantly differently in the subgroups, accompanied by significantly different prognosis, suggesting that our ten-gene signature can effectively identify the prognosis of patients. Thus, it will facilitate clinicians to make different treatment strategies. The DCA curve also implies that the nomogram constructed based on the ten-gene signature can benefit HNSCC patients at 1, 3, and 5 years.

Tumor immune microenvironment (TME) has a significant impact on tumor metastasis process and targeted therapy efficacy. We analyzed the proportion of 22 immune cell types in different subtypes. In the high-risk group with poor survival, the level of infiltration of activated Mast cells was significantly upregulated, suggesting its crucial role in the development of HNSCC. In addition, each of the seven risk genes, especially FN1, which had the highest correlation coefficient with Macrophage M0 and activated Mast cells. Therefore, FN1/activated Mast cells axis might be a interesting pathway.

Although our riskScore and the nomogram constructed based on it have better predictive performance, given the heterogeneity between cells, anoikis studies performed at the single-cell level may more accurately reflect the impact of ANRGs on the progression and prognosis of HNSCC patients. In addition, the limited amount of data in this study requires a larger sample size for the calibration of the prediction model.

In summary, our seven-gen model is able to well predict the survival in HNSCC patient, and the nomogram based on the model can help physicians develop personalized HNSCC treatments in clinical practice. Future studies on the molecular mechanisms associated with this feature and prospective randomized clinical trials will be clinically important and may provide a roadmap for precision medicine.

## Data Availability

Publicly available datasets were analyzed in this study. This data can be found here: The datasets analyzed in the current study are available in the TCGA repository (http://cancergenome.nih.gov/), GEO (https://www.ncbi.nlm.nih.gov/geo/). All raw data and original images can be found in the jianguoyun (https://www.jianguoyun.com/p/DevLgY4Q2arPChje7MgEIAA).
