# Supplementary figures and images for: A novel anoikis-related gene signature predicts prognosis in patients with head and neck squamous cell carcinoma and reveals immune infiltration
Source: Front Genet. 2022 Aug 26;13:984273. doi: 10.3389/fgene.2022.984273 (PMC9459093; doi:10.3389/fgene.2022.984273)

# Drug Sensitivity

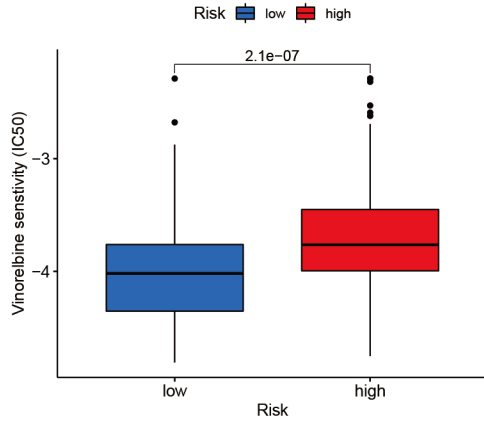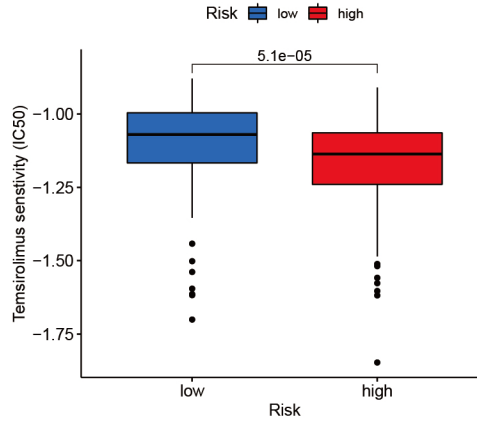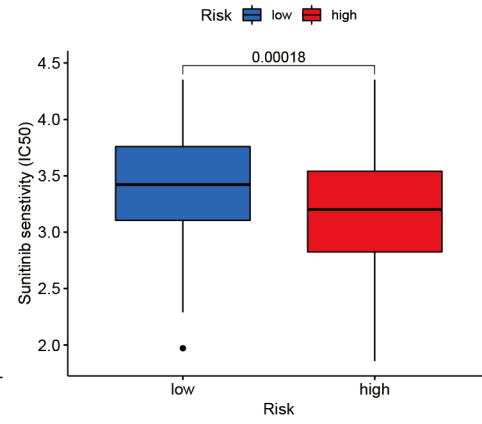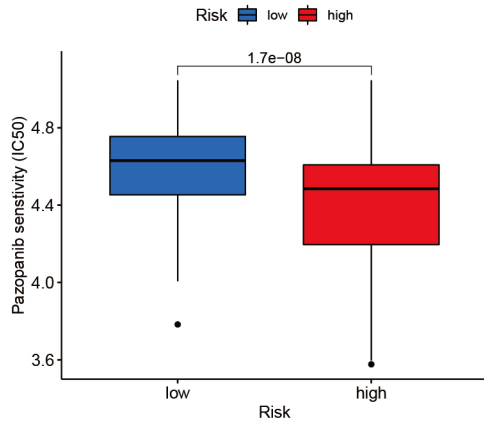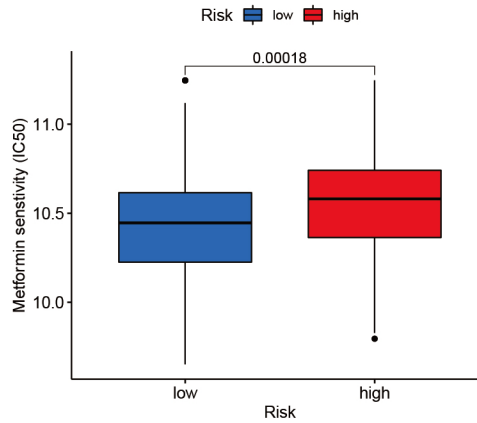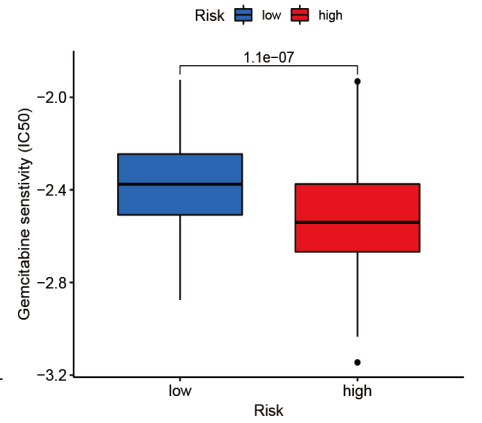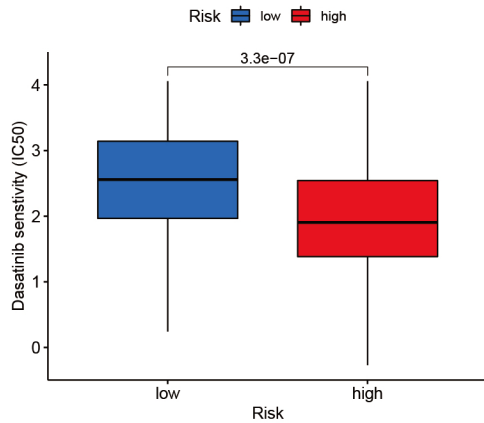

Supplement: Supplementary file 3 [file Image2.pdf]

KEGG enrichments in different clusters

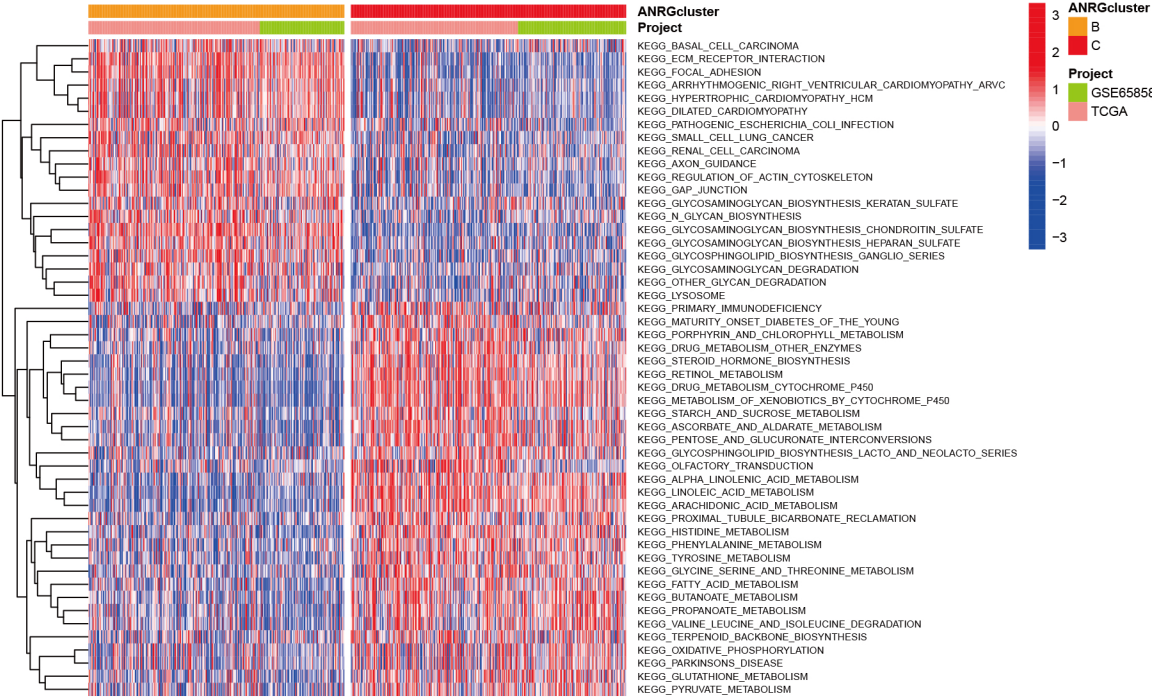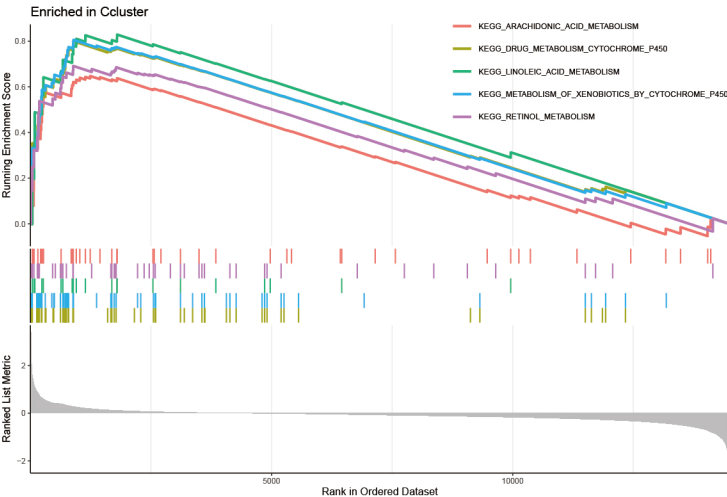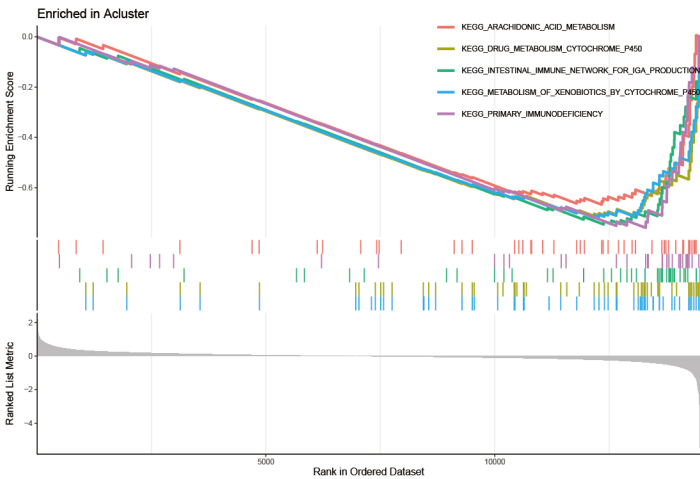

Supplement: Supplementary file 6 [file Image1.pdf]
