# Supplementary material for: A novel anoikis-related gene signature predicts prognosis in patients with head and neck squamous cell carcinoma and reveals immune infiltration
Source: Front Genet. 2022 Aug 26;13:984273. doi: 10.3389/fgene.2022.984273 (PMC9459093; doi:10.3389/fgene.2022.984273)
Supplement: Supplementary file 5 [file DataSheet1.docx]

All raw data and original images can be found in the jianguoyun ( <https://www.jianguoyun.com/p/DevLgY4Q2arPChje7MgEIAA> ).
